# One chiral fingerprint to find them all

**DOI:** 10.1186/s13321-024-00849-6

**Published:** 2024-05-13

**Authors:** Markus Orsi, Jean-Louis Reymond

**Affiliations:** Department of Chemistry, Biochemistry and Pharmaceutical Sciences, University of Bern, Freiestrasse 3, 3012 Bern, Switzerland

**Keywords:** Molecular fingerprints, Stereochemistry, Virtual screening, Chemical space, Atom-pairs

## Abstract

**Abstract:**

Molecular fingerprints are indispensable tools in cheminformatics. However, stereochemistry is generally not considered, which is problematic for large molecules which are almost all chiral.

Herein we report MAP4C, a chiral version of our previously reported fingerprint MAP4, which lists MinHashes computed from character strings containing the SMILES of all pairs of circular substructures up to a diameter of four bonds and the shortest topological distance between their central atoms. MAP4C includes the Cahn-Ingold-Prelog (CIP) annotation (*R*, *S*, *r* or *s*) whenever the chiral atom is the center of a circular substructure, a question mark for undefined stereocenters, and double bond cis–trans information if specified. MAP4C performs slightly better than the achiral MAP4, ECFP and AP fingerprints in non-stereoselective virtual screening benchmarks. Furthermore, MAP4C distinguishes between stereoisomers in chiral molecules from small molecule drugs to large natural products and peptides comprising thousands of diastereomers, with a degree of distinction smaller than between structural isomers and proportional to the number of chirality changes. Due to its excellent performance across diverse molecular classes and its ability to handle stereochemistry, MAP4C is recommended as a generally applicable chiral molecular fingerprint.

**Scientific contribution:**

The ability of our chiral fingerprint MAP4C to handle stereoisomers from small molecules to large natural products and peptides is unprecedented and opens the way for cheminformatics to include stereochemistry as an important molecular parameter across all fields of molecular design.

**Graphical Abstract:**

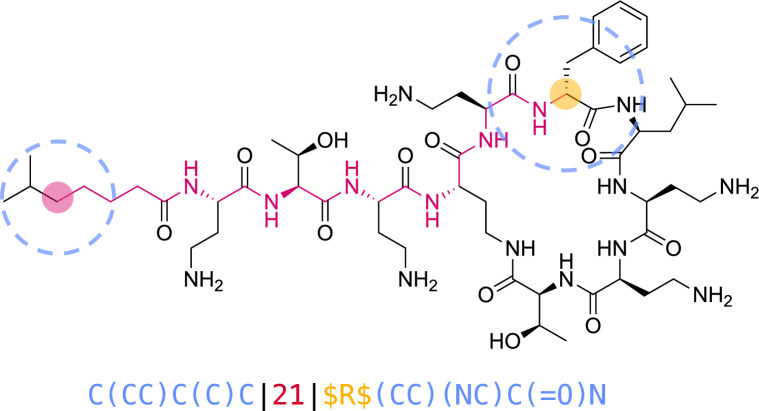

**Supplementary Information:**

The online version contains supplementary material available at 10.1186/s13321-024-00849-6.

## Introduction

Many computational tasks related to small molecule drug discovery, such as similarity searches [1, 2], target prediction [3–7], ligand-based virtual screening [8] and visualization of large databases of drug-like molecules [9–18], can be performed using vectors encoding molecular structure, called molecular fingerprints [19, 20]. Remarkably, molecular fingerprints work quite well to classify and compare bioactive molecules without considering stereochemical information, which is somewhat surprising considering that biological matter is essentially chiral and stereo-defined at the molecular level [21–23], but also reflects the fact one only rarely needs to distinguish between different stereoisomers of small molecule drugs, in part simply because many drug-like compounds are achiral.

In the context of developing computational tools for new modalities including beyond-Ro5 molecules [24, 25], in our case for peptides with variable chain topology and stereochemistry [26–28], we have adapted molecular fingerprints based on atom-pairs [29–32] for large molecules such as peptides and proteins [33–35]. In particular, we combined atom-pair analysis and circular substructures as encoded by the Morgan fingerprint ECFP4 [36, 37], with the principle of data compression using MinHashing [38–41], to design MAP4, a MinHashed Atom-Pair fingerprint. MAP4 encodes all possible pairs of circular substructures up to a diameter of four bonds in a molecule [42]. These pairs are written in the form of two canonicalized SMILES [43, 44] separated by the shortest topological distance, counted in bonds, between the corresponding pair of central atoms. Remarkably, MAP4 distinguishes molecular structures across different compound classes spanning from small molecules to natural products, peptides and the metabolome, for which other fingerprints such as the classical Morgan (ECFP4) [37] and Atom Pair (AP) [29] fingerprints fall short. In addition, MAP4 outperforms these and many other fingerprints in virtual screening benchmarks for both small molecule drugs [20] and peptides [42].

**Fig. 1:**
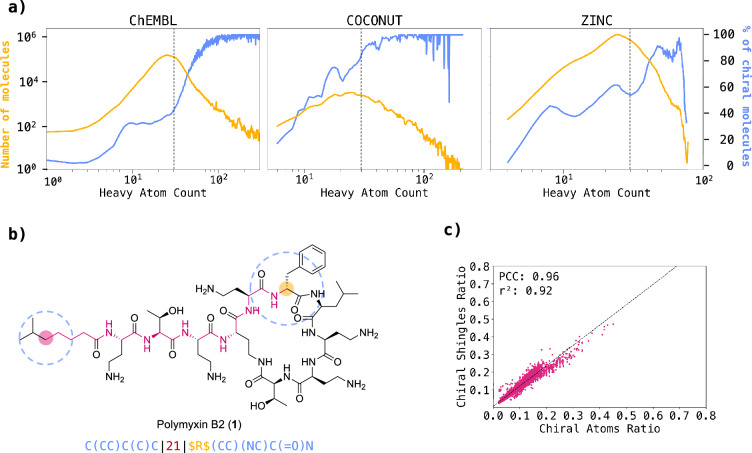
Molecular chirality and fingerprints. **a** Correlation between chirality and heavy atom count (HAC) across ChEMBL, COCONUT, and ZINC datasets. The blue line depicts the percentage of chiral molecules relative to HAC. A steady increase in the percentage of chiral molecules is observed with increasing HAC. The yellow line represents the total count of molecules corresponding to each HAC. **b** Chiral shingle generation concept exemplified on a selected atom pair of polymyxin B2. The generated shingle corresponds to the pair of circular substructures (blue) separated by the shortest topological distance (red) of their central atoms. Whenever the central atom of a substructure is chiral, the atom symbol in the substructure SMILES is replaced by the Cahn-Ingold-Prelog (CIP) descriptor (R, S, r, or s), or by a question mark (?) if the stereochemistry is not defined, bracketed by two “$” characters (yellow). **c** Percentage of molecular shingles containing chiral information vs. percentage of chiral atoms in the molecule for MAP4C (largest diameter of four bonds). These percentages were computed using a dataset of chiral molecules uniformly sampled from the Riniker & Landrum benchmark. The high r^2^ and Pearson correlation coefficients underscore a strong association between the two variables

Similarly to commonly used molecular fingerprints however, MAP4 does not include stereochemistry (cis–trans double bonds, enantiomers and diastereomers), which is clearly an omission considering that most molecules beyond Ro5, such as diverse natural products and synthetic compounds in the public databases ChEMBL [45], COCONUT [46], and ZINC [47], are chiral (Fig. 1a). To correct this omission and enable the cheminformatic analysis of compounds with multiple chiral centers such as carbohydrates and peptides, we now report MAP4C, an improved version of the MAP4 fingerprint. MAP4C includes the description of chiral centers following the Cahn-Ingold-Prelog (CIP) nomenclature in a fraction of molecular shingles (Fig. 1b, c), as well as double bond stereochemistry.

## Methods

### Fingerprint design

The chiral version of the MinHashed Atom-Pair fingerprint (MAPC) was implemented in Python using RDKit following these steps:

1. At every non-hydrogen atom, extract all circular substructures up to the specified maximum radius as isomeric, canonical SMILES. Isomeric information (“@” and “@@” characters) is manually removed from the extracted SMILES, while the implicit E/Z-isomerism (“/”, and “\” characters) are maintained. Allene chirality and conformational chirality such as in biaryls or in helicenes are not considered, as they cannot be specified in the SMILES notation. Radius 0 is skipped.
2. At the specified maximum radius, whenever the central atom of a circular substructure is chiral, replace the first atom symbol in the extracted SMILES with its Cahn-Ingold-Prelog (CIP) descriptor bracketed by two “$” characters ($CIP$). The CIP descriptor of the chiral atom is defined on the entire molecule, not on the extracted substructure.
3. At each radius, generate shingles for all possible pairs of extracted substructures. Each shingle contains two substructures and their topological distance in following format: “substructure 1 | topological distance | substructure 2”.
4. MinHash the list of shingles to obtain a fixed sized vector. The MinHashing procedure is explained in detail in our previous publication [38, 42].

### Benchmark

The virtual screening performance of the MAPC fingerprint was evaluated in a comparative study with commonly used fingerprints (ECFP4 [37], ECFP6 [37], Atom-Pair [29]) in a benchmark adapted from Riniker and Landrum [20]. Since the structure SMILES in the original benchmark do not contain any stereochemistry, the respective chiral SMILES (when applicable) were retrieved from the DUD [48], MUV [49] and ChEMBL [45] databases using the provided compound IDs.

Additional 60 peptide sets were included in the benchmark to test the performances of the fingerprints for large biomolecules. For each of 30 random linear sequences, a set containing 10,000 single-point mutants and a set containing 10,000 scrambled versions of the random sequence were generated and BLAST analogues labelled as actives. The precise generation procedure of the peptide datasets is described in our previous publication [42].

For every set, 5 randomly selected actives were extracted and stored in a separate file. The mean and standard deviation of pairwise ECFP4C Tanimoto and MAP4C Jaccard similarities of the five selected actives are reported in the Additional file 1: Figures S1, S2. Each of the selected actives was used as a query to rank the remaining compounds in the set based on fingerprint similarity (Jaccard similarity for MinHashed fingerprints; Dice similarity for folded fingerprints). AUC, EF1, EF5, BEDROC20, BEDROC100, RIE20 and RIE100 metrics were calculated for the obtained ranked lists and averaged along the 5 queries for every set in the benchmark. Additionally, the fingerprints were ranked based on the obtained performance metrics and finally the average rank of each fingerprint determined for all metrics. Pearson correlation coefficients and Friedman-Nemenyi post-hoc tests were calculated for all fingerprint pairs using the scipy and scikit-posthocs Python libraries.

### Stereoisomers, isomers and scrambled sequences

We enumerated all possible stereoisomers of molecules **1–14** (Figs. 1c and  4) by generating all possible isomeric SMILES combinations, canonicalizing them, and removing duplicates. We additionally enumerated all possible permutations of ln65 (**7**) and polymyxin B2 (**1**) sequences, obtaining a total of 330 and 1,512 scrambled sequences respectively. Structural isomers of 1,4-diaminocyclohexane (**15)** and aminopiperazine (**16)** were extracted from GDB-13 using the MQN-browser [50, 51]. The extracted sets contained 203 structural isomers of **15,** of which 156 contained one or more stereocenters and 48 structural isomers of **16,** of which 29 contained one or more stereocenters. For each structural isomer, all possible stereoisomers were generated using the RDKit “EnumerateStereoisomers” function, yelding 746 unique structures for **15** and 126 for **16**. For all stereoisomers and permutations, fingerprints were calculated as 2048-bit vectors.

### TMAP

The indices obtained from the MAP4C calculation were used to create a locality-sensitive hashing (LSH) forest of 32 trees. For each molecular structure, the 500 approximate nearest neighbors in the MAP4C feature space were extracted from the LSH forest and used to calculate the TMAP layout [16]. The resulting layout was displayed in an interactive TMAP using the open-source Faerun package [15].

## Results and discussion

### Encoding stereochemistry in MAP fingerprints

The MAP (MinHashed Atom-Pair) fingerprint of a molecule consists in a series of MinHashes computed from the list of its molecular shingles [38–41]. A molecular shingle is written for each possible pair of circular substructures of a given diameter (2 bonds for MAP2, 4 bonds for MAP4, 6 bonds for MAP6), written as canonicalized SMILES, separated by the shortest topological distance separating the central atoms, counted in bonds [42]. We preserve the *Z/E* double bond information in all shingles whenever the entire double bond is included in a shingle. To encode stereocenter information into our fingerprints, we label chiral atoms with their Cahn–Ingold–Prelog (CIP) descriptor (*R*,* S*, *r* or *s*), as computed by RDKit, whenever stereochemistry is defined, or label them with a question mark (“?”) if stereochemistry is not specified. Importantly, we only apply the chiral label when a chiral atom is the central atom of a circular substructure and only for shingles with the largest diameter considered. The concept is illustrated for one of the possible pairs involving the stereocenter in polymyxin B2 (**1**, Fig. 1b).

When applied to a dataset of chiral molecules uniformly sampled from the Riniker and Landrum benchmark (Additional file 1: Figure S3) [20], we find that the percentage of molecular shingles containing chiral information is approximately the same as the percentage of chiral atoms in a molecule for MAP2C (largest diameter of two bonds, Additional file 1: Figure S4a), MAP4C (largest diameter of four bonds, Fig. 1c) and MAP6C (largest diameter of six bonds, Additional file 1: Figure S4b). Most importantly, chiral information only appears in a relatively small fraction of all possible shingles, such that any defined stereoisomer of a molecule has a relatively high similarity to the molecule without assigned stereochemistry, for which the MAPC fingerprint is identical to the MAP fingerprint.

### Virtual screening benchmark

The relevance of any molecular fingerprint for drug discovery can be tested by attempting to retrieve known bioactive compounds for a given target by nearest-neighbor searches from one of the known active compounds in a dataset in which the known actives have been mixed with so-called decoys. These decoys are molecules selected randomly from databases to have similar physico-chemical properties as the actives, but which are not documented to be active on the target. Here we used the reference benchmarking dataset of Riniker and Landrum for small molecule drugs [20], which considers 118 active and decoy datasets taken from DUD [48], MUV [49], and ChEMBL [45]. For larger molecules, we used our previously reported set of 60 different randomly chosen 10−, 15− and 20−mer peptides mixed with either random single point mutants (30 sets), or sequence scrambled analog (30 sets) [42], for which we challenge the fingerprint to retrieve BLAST search analogs [52].

**Fig. 2:**
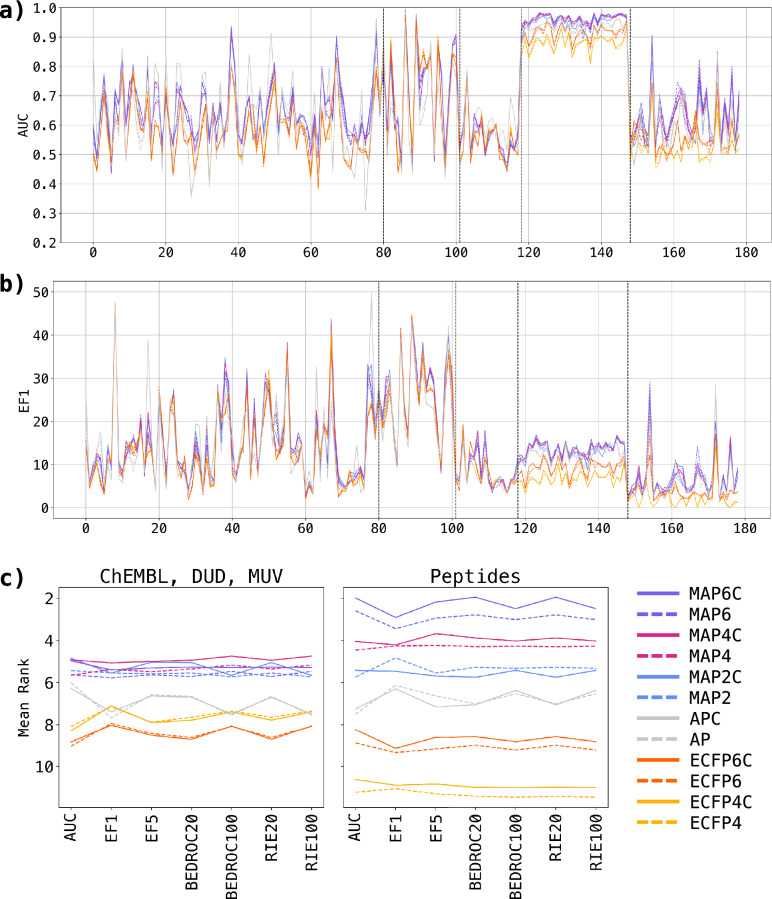
Virtual Screening benchmark (**a**) AUC and (**b**) EF1 of MAP6 (purple), MAP4 (magenta), MAP2 (blue), AP (grey), ECFP6 (orange) and ECFP4 (yellow) and across all small molecules and peptide targets (80 ChEMBL targets, 21 DUD targets, 17 MUV targets, 30 mutated peptide targets, and 30 scrambled peptide targets). Chiral fingerprints are displayed as bold lines, non-chiral fingerprints are displayed as dashed lines. The value displayed for each dataset is the mean metric of 5 runs. **c** Mean ranks of fingerprints across all virtual screening datasets for each metric. Small molecule sets (ChEMBL, DUD, MUV) and peptide sets are presented separately to highlight the differences in relative performance

We compared the performance of MAP2C, MAP4C, and MAP6C with their respective achiral counterparts, as well as with reference binary fingerprints ECFP4, ECFP6, and AP, and their corresponding chiral versions (ECFP4C, ECFP6C, and APC). The primary objective of the benchmark experiment was to ensure that the inclusion of chirality does not compromise the baseline virtual screening capabilities of the original MAP fingerprint. Indeed, fingerprints in their chiral and non-chiral versions demonstrated comparable performances across various test sets and performance metrics, showing that including chirality information was not detrimental to fingerprint performance in these benchmarks (Fig. 2a, b and Additional file 1: Figure S5–S9).

The ranks of the different fingerprints for the various performances measures showed that the MAP fingerprints performances were slightly ahead of the other fingerprints, with MAP4C appearing with the best ranks in the small molecule benchmark and MAP6C in the peptide benchmark (Fig. 2c). However, a pairwise Friedman-Nemenyi test across all performance metrics showed that the difference between chiral over non-chiral fingerprints of each type (MAPC vs. MAP, ECFPC vs. ECFP and APC vs AP) was not significant (Additional file 1: Figures S10-16). The only statistically significant differences were between groups. For instance, MAP(C) fingerprints significantly outperformed ECFP(C) and AP(C) fingerprints with exception of AP(C) for the AUC metric. MAP(C) fingerprints combine high local precision of circular substructure encoding, akin to ECFPs, with the perception of atom pairs reflecting global structural features, akin to AP fingerprints. This combination is particularly effective in scenarios where both local precision and global structure are relevant to differentiate between active and non-active molecules, possibly explaining the higher performance of the MAP(C) fingerprints compared to ECFP(C) and AP(C).

### Finding all stereoisomers

**Fig. 3:**
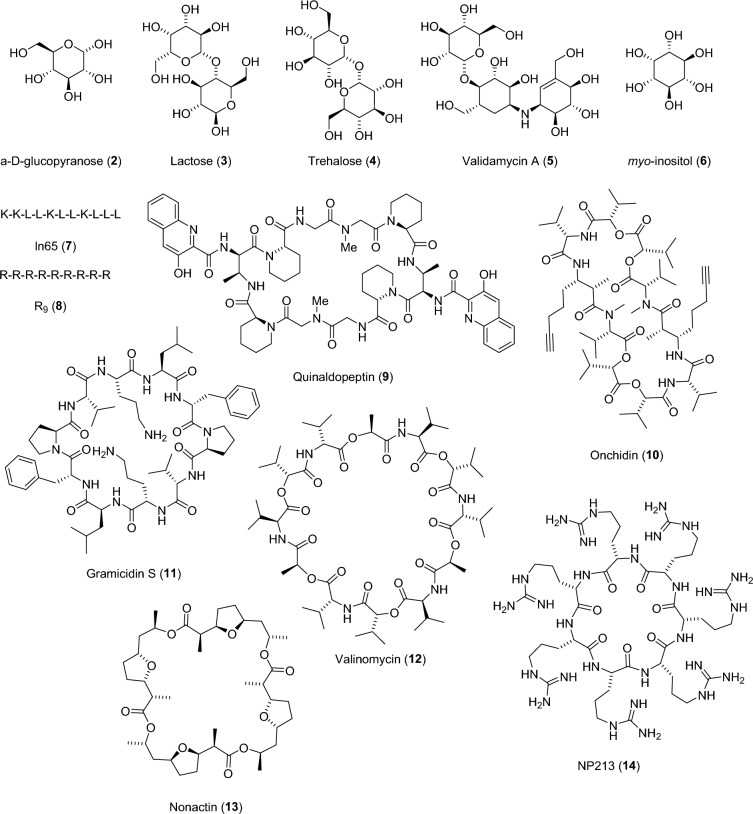
Structures of natural products and peptides selected for the stereoisomer distinction task

In addition to be on par with non-chiral fingerprints for the above virtual screening benchmarks, one would expect a chiral fingerprint to distinguish all possible stereoisomers of a chiral molecule. To test the chiral differentiation of our fingerprints, we investigated their ability to assign a different fingerprint value for each stereoisomer on a series of stereochemically complex molecules comprizing carbohydrates, peptides and macrocyclic natural products containing up to thousands of stereoisomers per molecule (Fig. 3 and Table 1).

**Table 1:** Stereoisomer and scrambled sequence distinction task for selected natural products and peptides with multiple chiral centers and varying degrees of internal symmetry

Query^a)^	N / Sym.^b)^	Total^c)^	MAP6C	MAP4C	MAP2C	APC	ECFP6C	ECFP4C
α-D-glucopyranose (**2**)	5 /–	32	32	32	32	11	32	32
Lactose (**3**)	10 / –	1,024	1,024	1,024	992	443	1,024	1,024
Trehalose (**4**)	10 / C_2_	528	528	528	516	336	528	512
Validamycin A (**5**)	14 / –	16,384	16,384	16,384	16,384	7,657	16,384	16,384
Inositol (**6**)	6 / C_6v_	9	9	9	9	1	1	1
ln65 (**7**)	11 / –	2,048	2,048	2,048	2,048	196	1,140	36
ln65 (scrambled)	11 / –	330	330	330	330	330	8	4
ln65 (dia × scrambled)	11 / –	675,840	675,840	675,840	675,840	90,217	38,500	144
R_9_ (**8**)	9 / –	512	512	512	512	146	88	12
Polymyxin B2 (**1**)^d)^	12 / –	4,096	4,096	4,096	4,096	2,500	4,096	1,536
PMB2 (scrambled)^e)^	9 / –	1,512	1,512	1,512	1,512	1,512	861	75
PMB2 (dia × scrambled)^f)^	9 / –	774,144	774,144	774,144	774,144	287,631	602,003	9,312
PMB2 (*R, S* or undefined)	12 / –	531,441	531,441	531,441	531,441	277,901	531,441	137,781
Quinaldopeptin (**9**)	8 / C_2_	136	136^ g)^	136	134	64	132	90
Onchidin (**10**)	12 / C_2_	2,080	2,080	2,080	2,064	469	1,760	810
Gramicidin S (**11**)	10 / C_2_	528	528	504	334	25	448	243
Valinomycin (**12**)	12 / C_3_	1,376	1,250	714	416	112	616	27
Nonactin (**13**)	16 / C_4_	16,456	16,425	16,176	10,045	13,189	6,474	675
NP213 (**14**)	7 / C_7_	20	7	13	17	13	5	3

^a)^Name and nr. of molecule. See Fig. 4 for structural formulae

^b)^N = number of stereocenters in the molecule. *Sym* rotational molecular symmetry for the molecule without chiral labels

^c)^Number of possible stereoisomers considering inversion of all chiral centers in the molecule and the internal symmetry, or number of sequence isomers (scrambled). The number of different fingerprint values for each fingerprint type is given in the following columns. All fingerprint were used with 2,048 bit size unless otherwise noted

^d)^all stereocenters in the molecule are considered

^e)^amino acids are scrambled, the *N*-terminal fatty acid and the branching Dab residue are maintained

^f)^only the α-carbon chirality of the scrambled residues was considered here, which corresponds to 512 stereoisomers per scrambled sequence

^g)^with 4,096 bits, only 135 different FP values are obtained with 2,048 bits due to a bit collision

**Fig. 4:**
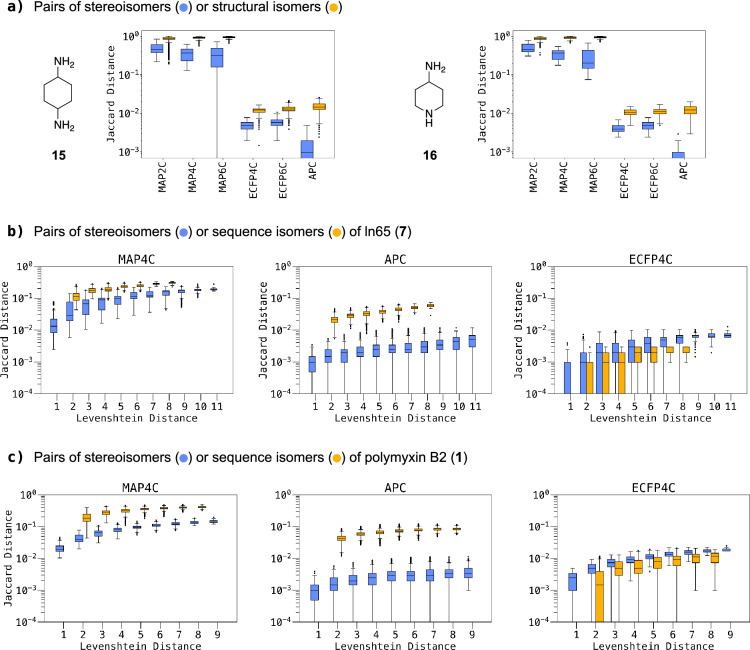
Differentiation between stereoisomers and structural isomers, shown as box plots of average Jaccard distances between pairs of stereoisomers (blue) or structural/sequence isomers (yellow). **a** structural isomers of 1,4-diaminocyclohexane (203) and 4-aminopiperidine (48) and their diastereomers. The skewed distribution of Jaccard distance of **15** with MAP6C is caused by two outliers exhibiting a distance of 0 which cannot be represented on the log scale and is likely due to a bit-clash issue. **b** sequence isomers (330) or diastereomers (2,048) of ln65 (**7**) as function of the Levenshtein distance separating each pair. **c** sequence isomers (1,512) or diastereomers (512) of polymyxin B2 (**1**) as function of the Levensthein distance separating each pair. See Figures S10 and S11 for plots with MAP6C, MAP2C and ECFP6C. See methods for details

For carbohydrates, both MAP6C and MAP4C readily distinguished the 32 stereoisomers of α-D-glucopyranose (**2**), the 1024 stereoisomers of the disaccharide lactose (**3**), the 528 possible stereoisomers of the non-reducing, C_2_-symmetrical α-diglucoside trehalose (**4**), the 16,384 stereoisomers of the aminoglycoside antibiotic validamycin A (**5**), and the nine possible stereoisomers of the signaling carbocyclic sugar *myo*-inositol (**6**). By contrast, the four other chiral fingerprints tested all fell short in at least one of the six cases, and APC failed on all of them.

Our MinHashed fingerprints performed very well with peptide stereoisomers. In the case of the antimicrobial undecapeptide ln65 (**7**), a membrane disruptive antimicrobial peptide whose activity/toxicity balance is modulated by stereochemical variations and which motivated the present study [28], the three chiral MAP fingerprints distinguished all the 2,048 possible stereoisomers. By contrast, ECFP6C only saw about half of them and ECFP4C and APC distinguished less than 10%, most likely because this peptide is composed of only lysine and leucine residues, which reduces the number of possible substructures. The chiral MAP fingerprints also distinguished the 330 possible sequence-scrambled isomers of **7** and the 675,840 possible stereoisomers of sequence-scrambled isomers of **7**. By comparison, APC succeeded for the 330 scrambled sequences but failed on the larger set, and both chiral ECFPs failed in both cases, which can be attributed to the absence of long-range substructures in ECFP fingerprints.

The ability of chiral MAP fingerprints to perceive peptide stereoisomers was also well illustrated by their ability to distinguish all 512 stereoisomers of the cell-penetrating peptide nona-arginine (**8**) [53, 54], as well as the 4096 stereoisomers of polymyxin B2 (**1**), used as last resort antibiotic against multidrug resistant bacteria [55]. In the latter case, our fingerprints also distinguished between the 1,512 possible sequence-scrambled isomers of **1**, the 774,144 possible sequence-scrambled stereoisomers of **1**, as well as between the 531,441 possible assignments of chirality as *R, S*, or undefined stereochemistry in the 12 chiral centers of **1**. An undefined stereochemistry corresponds to a stereorandomized position accessible by chemical synthesis using a racemic amino acid at that position (stereorandomization at multiple position can lead to partially active analogs as reported for **1**) [56]. In all of these cases, APC and ECFPCs were unable to distinguish all possibilities.

Macrocyclic natural products with rotational symmetries were particularly challenging for chiral fingerprints. For instance, only MAP4C and MAP6C correctly identified the 136 possible stereoisomers of the cyclic peptide antibiotic quinaldopeptin (**9**) and the 2,080 stereoisomers of the cytotoxic macrocyclic depsipeptide onchidin (**10**), two natural product macrocycles with C_2_ symmetry. By contrast, the 528 stereoisomers of the C_2_ symmetrical antimicrobial macrocyclic peptide gramicidin S (**11**) were only distinguished by MAP6C. Furthermore, none of the chiral fingerprints tested was able to cope with the C_3_ symmetrical dodecadepsipeptide antibiotic valinomycin (**12**, 1,376 stereoisomers), the C_4_ symmetrical macrolide ionophore antibiotic nonactin (**13**, 16,456 stereoisomers), or the C7 symmetrical hepta-arginine cyclic peptide NP213 developed as antifungal agent (**14**, 20 stereoisomers). Note that all fingerprints were used with 2,048-bits, but that performance did not increase significantly when using much larger bit sizes or without MinHashing or folding.

### Ranking stereoisomers versus isomers

The degree of differentiation between stereoisomers should be proportional to the number of stereochemical changes between any two stereoisomers, and should also be smaller than the difference to a different molecule such as a structural isomer. We tested the ability of our chiral fingerprints for this task for small and large molecules separately. As a test case for small molecules, we computed Jaccard distances between all pairs involving the 203 structural isomers of 1,4-diaminocyclohexane (**15**), a ring fragment which is enriched in bioactive molecules from ChEMBL [57, 58], and between all pairs of stereoisomers in the set. We similarly analyzed all pairs involving the 48 structural isomers of 4-aminopiperazine (**16**), a similar drug scaffold, and the stereoisomeric pairs within the set. Generally, MAPC distances were higher than those of other fingerprints. This outcome is unsurprising, given that MAPC encodes a notably greater number of features, which also contributes to its high precision. In both test cases, all six fingerprints ranked pairs stereoisomers closer to each other than pairs of structural isomers (Fig. 4a**/**b).

**Fig. 5:**
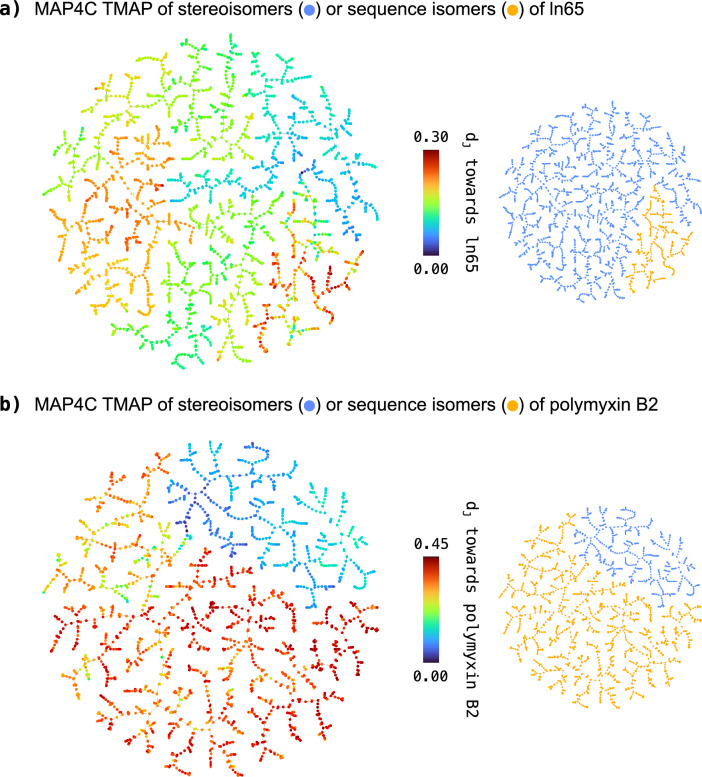
MAP4C TMAPs showing the Jaccard distance (d_J_; rainbow) of stereoisomers (blue) and sequence isomers (yellow) towards their respective queries: (**a**) ln65, 2,048 diastereomers and 330 sequence isomers. The interactive version of the TMAP is accessible under https://tm.gdb.tools/map4/MAP4C_ln65/ (**b**) polymyxin B2, 512 diastereomers and 1,512 sequence isomers. The interactive version of the TMAP is accessible under https://tm.gdb.tools/map4/MAP4C_pmb2/

For peptides, we measured Jaccard distances between pairs of scrambled-sequence isomers versus pairs of stereoisomers with the same sequence for ln65 (**7**) and polymyxin B2 (**1**). For peptides, the degree of sequence similarity can also be measured by the Levenshtein distance, which represents the minimum number of mutations necessary to transform one sequence into another one, considering residue type changes, stereochemical inversions, insertions and deletions (Fig. 4c**/**d and Additional file 1: Figure S17, S18). Jaccard distances generally increased with increasing Levensthein distances for all fingerprints. Similar to small molecules, distances between peptide stereoisomers were smaller than between sequence isomers only for chiral MAP fingerprints and APC. However, chiral ECFPs assigned larger distances to stereoisomers than to sequence isomers, which probably relates to their inability to distinguish many pairs of sequence isomers. For both ln65 (**7**) and polymyxin B2 (**1**), the lower Jaccard distances between stereoisomers compared to sequence isomers was well visible in TMAP representations of each dataset constructed using MAP4C as similarity measure (Fig. 5a**/**b) [16]. In both cases, there was a complete separation between the 2,048/512 stereoisomers of the parent peptide and the 330/1,512 sequence isomers.

## Conclusions

In summary, the data above shows that the chiral versions of MAP fingerprints reported here perform as good as their achiral versions in non-stereoselective virtual screening benchmarks. Remarkably, our chiral MAP fingerprints are able to distinguish stereoisomers even in cases involving up to thousands of stereoisomers where the chiral versions of ECFP and AP do not perform well. Furthermore, the chiral MAP Jaccard distances between enantiomers or stereoisomers are generally shorter than for structural isomers, allowing to use chiral MAP fingerprints as a refinement of their achiral version. Because MAP4C computes faster than MAP6C due to the small number of atom pairs considered, we recommend MAP4C as the molecular fingerprint of choice for comparing molecules spanning from small drug-like building blocks to large natural products and peptides. The ability of our chiral fingerprint MAP4C to handle stereoisomers from small molecules to large natural products and peptides is unprecedented and opens the way for cheminformatics to include stereochemistry as an important molecular parameter across all fields of molecular design.

### Supplementary Information

**Additional file 1: Figure S1.** Mean and standard deviation of the pairwise ECFP4C similarities calculated for all 5 selected actives of each dataset contained in the benchmarking platform. **Figure S2.** Mean and standard deviation of the pairwise MAP4C similarities calculated for all 5 selected actives of each dataset contained in the benchmarking platform. **Figure S3.** Property distribution in the set uniformly sampled from the extended benchmark. **Figure S4.** Scatterplots of chiral shingle ratio vs. chiral atoms ratio. **Figure S5.** EF5 values across all small molecules and peptide targets**. Figure S6.** BEDROC20 values across all small molecules and peptide targets. **Figure S7.** BEDROC100 values across all small molecules and peptide targets. **Figure S8.** RIE20 valuesbvacross all small molecules and peptide targets. **Figure S9.** RIE100 values across all small molecules and peptide targets. **Figure S10.** Pairwise Pearson correlations and Friedman-Nemenyi test among tested fingerprints, based on the ranked AUCs. **Figure S11.** Pairwise Pearson correlations and Friedman-Nemenyi test among tested fingerprints, based on the ranked EF1s from benchmark datasets. **Figure S12.** Pairwise Pearson correlations and Friedman-Nemenyi test among tested fingerprints, based on the ranked EF5s from benchmark datasets. **Figure S13.** **a) **Pairwise Pearson correlations and Friedman-Nemenyi test among tested fingerprints, based on the ranked BEDROC20s from benchmark datasets. **Figure S14.** Pairwise Pearson correlations and Friedman-Nemenyi test among tested fingerprints, based on the ranked BEDROC100s from benchmark datasets. **Figure S15.** Pairwise Pearson correlations andFriedman-Nemenyi test among tested fingerprints, based on the ranked RIE20s from benchmark datasets. **Figure S16.** Pairwise Pearson correlations and Friedman-Nemenyi test among tested fingerprints, based on the ranked RIE100s from benchmark datasets. **Figure S17.** Comparative analysis of MAP2C, MAP4C, MAP6C, APC, ECFP4C and ECFP6C Jaccard distance assignment on ln65 diastereomers and structural isomers. **Figure S18.** Comparative analysis of MAP2C, MAP4C, MAP6C, APC, ECFP4C and ECFP6C Jaccard distance assignment on polymyxin B2 diastereomers and structural isomers.

## Data Availability

The source codes and datasets used for this study are available at https://zenodo.org/records/10389905 The code for MAPC can be found at https://github.com/reymond-group/mapchiral.

## Abbreviations

AP(C): Atom pair fingerprint (chiral)
AUC: Area under the curve
BEDROC: Boltzmann-enhanced discrimination of the receiver operating characteristic
BLAST: Basic local alignment search tool
CIP: Cahn-Ingold-Prelog
DUD: Directory of useful decoys
ECFP(C): Extended connectivity fingerprint (chiral)
EF: Enrichment factor
FP: Fingerprint
GDB: Generated database
HAC: Heavy atom count
JD: Jaccard distance
LSH: Locality sensitive hashing
MAP(C): MinHash atom pair fingerprint (chiral)
MQN: Molecular quantum numbers
MUV: Maximum unbiased validation data sets
PMB2: Polymyxin B2
RIE: Robust initial enhancement
Ro5: Rule of fives
ROC: Receiver operating characteristic
SMILES: Simplified molecular-input line-entry system
TMAP: Tree map
