# Supplementary material for: One chiral fingerprint to find them all
Source: J Cheminform. 2024 May 13;16:53. doi: 10.1186/s13321-024-00849-6 (PMC11090803; doi:10.1186/s13321-024-00849-6)
Supplement: Supplementary file 1 — Additional file 1: Figure S1. Mean and standard deviation of the pairwise ECFP4C similarities calculated for all 5 selected actives of each dataset contained in the benchmarking platform. Figure S2. Mean and standard deviation of the pairwise MAP4C similarities calculated for all 5 selected actives of each dataset contained in the benchmarking platform. Figure S3. Property distribution in the set uniformly sampled from the extended benchmark. Figure S4. Scatterplots of chiral shingle ratio vs. chiral atoms ratio. Figure S5. EF5 values across all small molecules and peptide targets. Figure S6. BEDROC20 values across all small molecules and peptide targets. Figure S7. BEDROC100 values across all small molecules and peptide targets. Figure S8. RIE20 valuesbvacross all small molecules and peptide targets. Figure S9. RIE100 values across all small molecules and peptide targets. Figure S10. Pairwise Pearson correlations and Friedman-Nemenyi test among tested fingerprints, based on the ranked AUCs. Figure S11. Pairwise Pearson correlations and Friedman-Nemenyi test among tested fingerprints, based on the ranked EF1s from benchmark datasets. Figure S12. Pairwise Pearson correlations and Friedman-Nemenyi test among tested fingerprints, based on the ranked EF5s from benchmark datasets. Figure S13. a) Pairwise Pearson correlations and Friedman-Nemenyi test among tested fingerprints, based on the ranked BEDROC20s from benchmark datasets. Figure S14. Pairwise Pearson correlations and Friedman-Nemenyi test among tested fingerprints, based on the ranked BEDROC100s from benchmark datasets. Figure S15. Pairwise Pearson correlations andFriedman-Nemenyi test among tested fingerprints, based on the ranked RIE20s from benchmark datasets. Figure S16. Pairwise Pearson correlations and Friedman-Nemenyi test among tested fingerprints, based on the ranked RIE100s from benchmark datasets. Figure S17. Comparative analysis of MAP2C, MAP4C, MAP6C, APC, ECFP4C and ECFP6C Jaccard distance assignme [file 13321_2024_849_MOESM1_ESM.pdf]

Supplementary Information for:
One chiral fingerprint to find them all

Markus Orsi[a] and Jean-Louis Reymond[a]*

[a)] Department of Chemistry, Biochemistry and Pharmaceutical Sciences, University of Bern,

Freiestrasse 3, 3012 Bern, Switzerland

e-mail: jean-louis.reymond@unibe.ch

Table of Contents

1. Supplementary figures

Figure S1 2
Figure S2 2
Figure S3 3
Figure S4 3
Figure S5 4
Figure S6 4
Figure S7 5
Figure S8 5
Figure S9 6
Figure S10 7
Figure S11 8
Figure S12 9
Figure S13 10
Figure S14 11
Figure S15 12
Figure S16 13
Figure S17 14
Figure S18 15

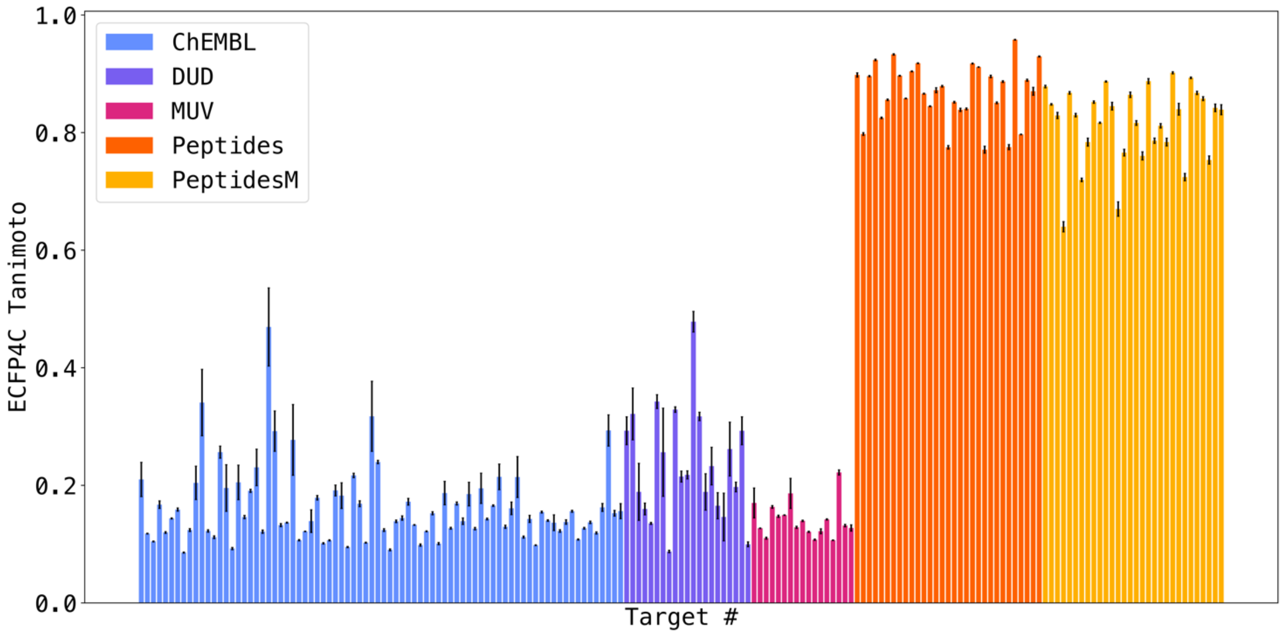

Figure S1: Mean and standard deviation of the pairwise similarities calculated for all 5 selected actives of each dataset contained in the benchmarking platform. Actives are encoded using the chiral ECFP4 (radius=2, nBits=2048) fingerprint and Tanimoto similarities determined for all possible pairs. ChEMBL, DUD and MUV sets comprise the original Riniker & Landrum benchmark. The “Peptides” set contains scrambled sequences of the same peptide. The “PeptidesM” set contains single point mutants of the same peptide.

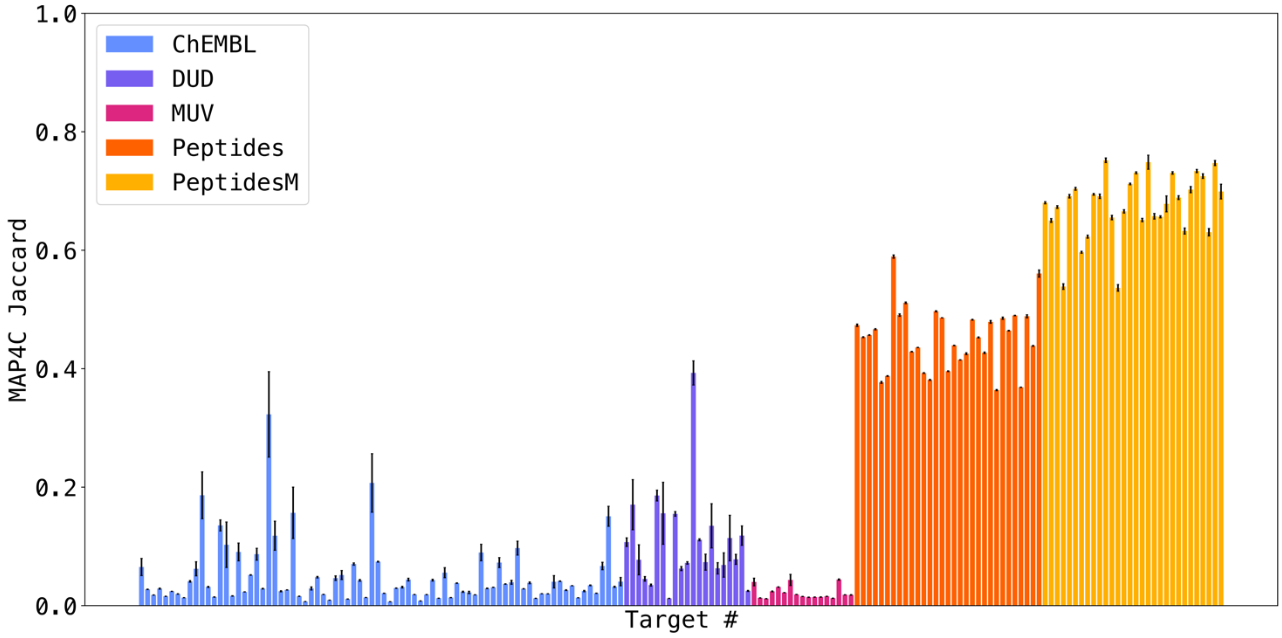

Figure S2: Mean and standard deviation of the pairwise similarities calculated for all 5 selected actives of each dataset contained in the benchmarking platform. Actives are encoded using the MAP4C (max_radius=2, n_permutations=2048) fingerprint and Jaccard similarities determined for all possible pairs. ChEMBL, DUD and MUV sets comprise the original Riniker & Landrum benchmark. The “Peptides” set contains scrambled sequences of the same peptide. The “PeptidesM” set contains single point mutants of the same peptide.

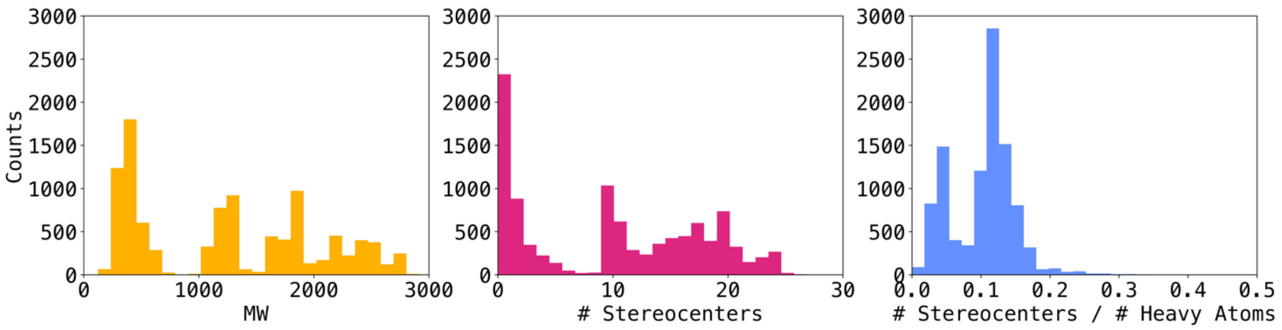

Figure S3. Distribution of molecular weight (MW) (yellow), number of stereocenters (magenta) and ratio of stereocenters to heavy atom count (blue) in the set uniformly sampled from the extended benchmark. The set contained a total of 10,122 compounds and was used to determine the relative impact of stereochemistry encoding on total similarity.

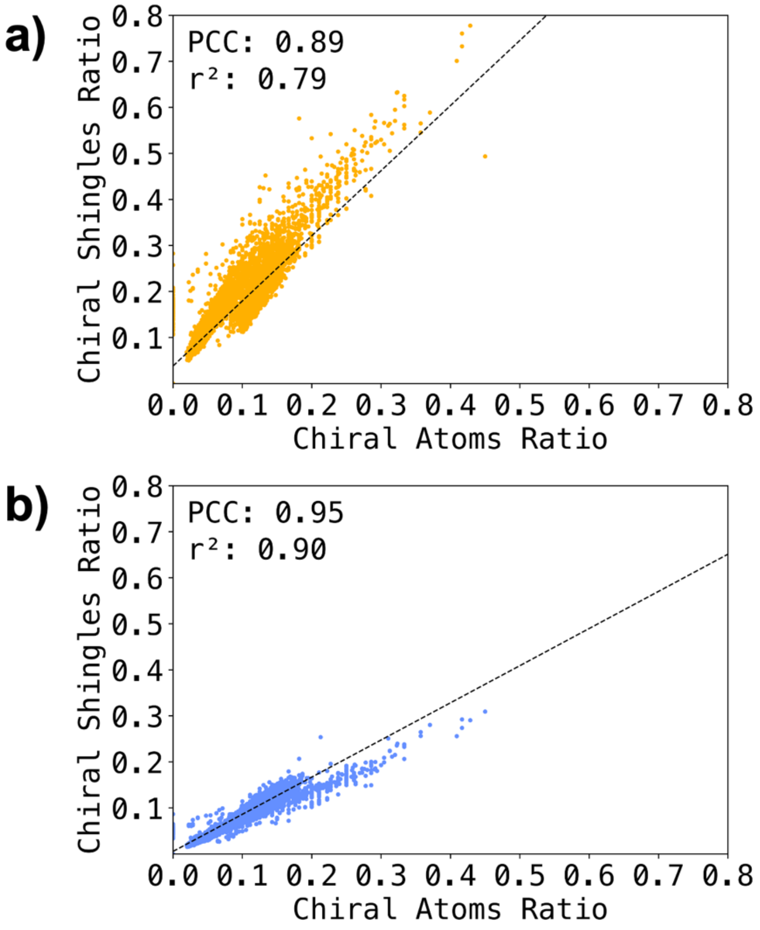

Figure S4. Scatterplots of chiral shingle ratio vs. chiral atoms ratio for a) radius = 1 b) radius = 2 and c) radius = 3. Additionally, the r^2 of the linear fit and the Pearson correlation coefficient (PCC) are reported. All reported PCCs are statistically significant.

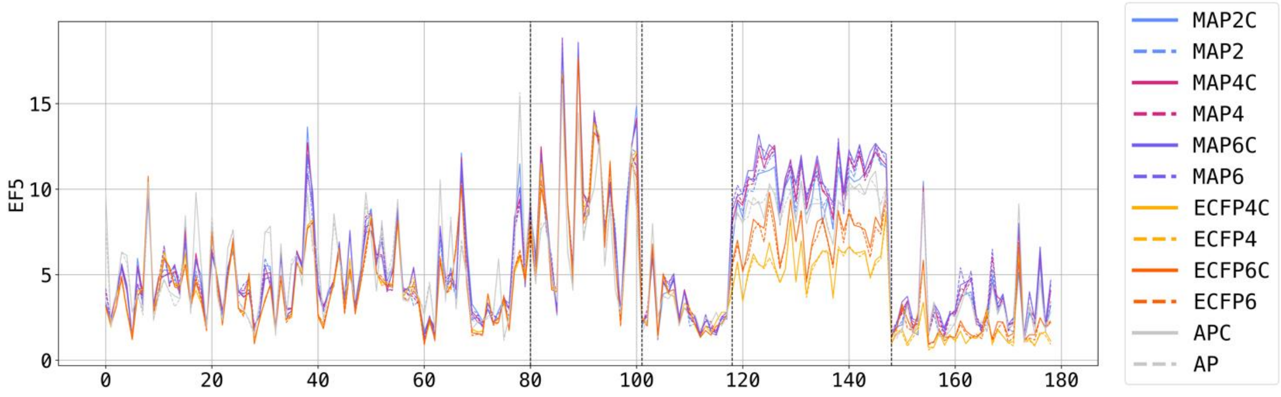

Figure S5. EF5 of MAP2 (blue), MAP4 (magenta), MAP6 (purple), AP (grey), ECFP4 (yellow) and ECFP6 (orange) across all small molecules and peptide targets (80 ChEMBL targets, 21 DUD targets, 17 MUV targets, 30 mutated peptide targets, and 30 scrambled peptide targets). Chiral fingerprints are displayed as bold lines, non-chiral fingerprints are displayed as dashed lines. The value displayed for each dataset is the mean metric of 5 runs.

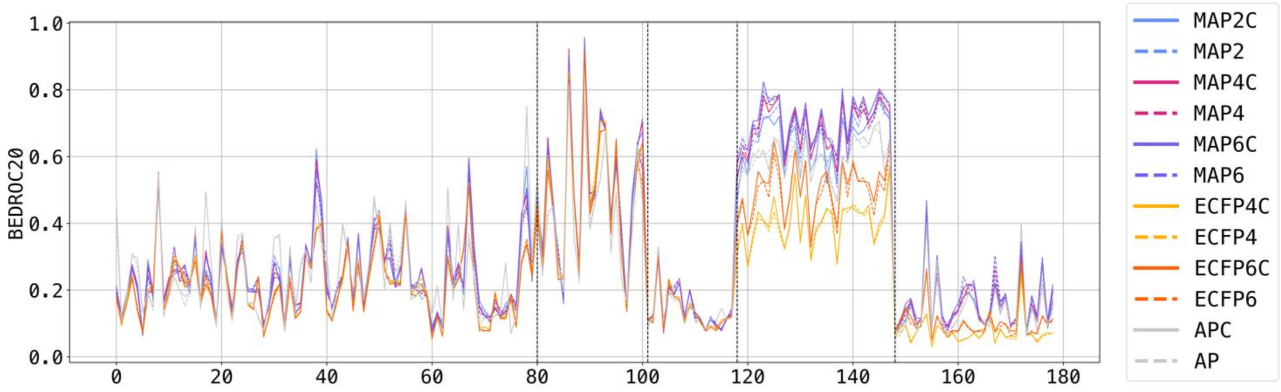

Figure S6. BEDROC20 of MAP2 (blue), MAP4 (magenta), MAP6 (purple), AP (grey), ECFP4 (yellow) and ECFP6 (orange) across all small molecules and peptide targets (80 ChEMBL targets, 21 DUD targets, 17 MUV targets, 30 mutated peptide targets, and 30 scrambled peptide targets). Chiral fingerprints are displayed as bold lines, non-chiral fingerprints are displayed as dashed lines. The value displayed for each dataset is the mean metric of 5 runs.

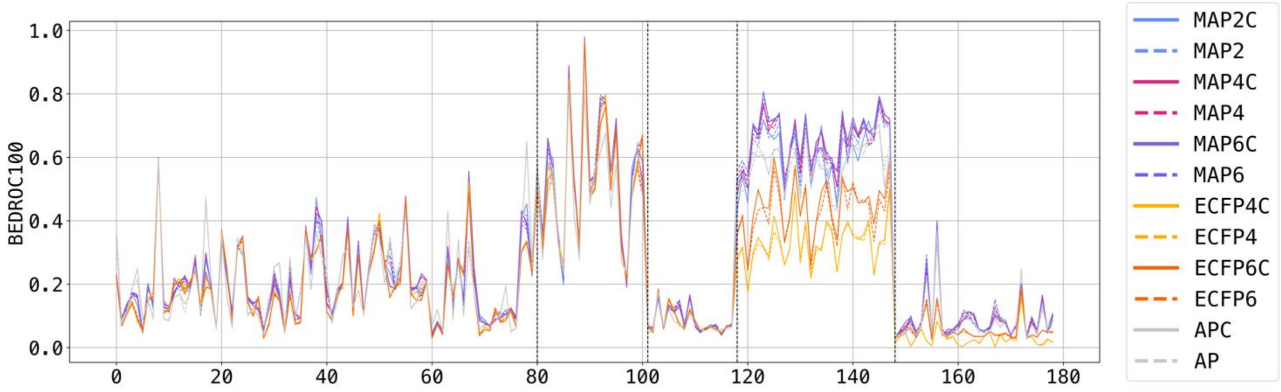

Figure S7. BEDROC100 of MAP2 (blue), MAP4 (magenta), MAP6 (purple), AP (grey), ECFP4 (yellow) and ECFP6 (orange) across all small molecules and peptide targets (80 ChEMBL targets, 21 DUD targets, 17 MUV targets, 30 mutated peptide targets, and 30 scrambled peptide targets). Chiral fingerprints are displayed as bold lines, non-chiral fingerprints are displayed as dashed lines. The value displayed for each dataset is the mean metric of 5 runs.

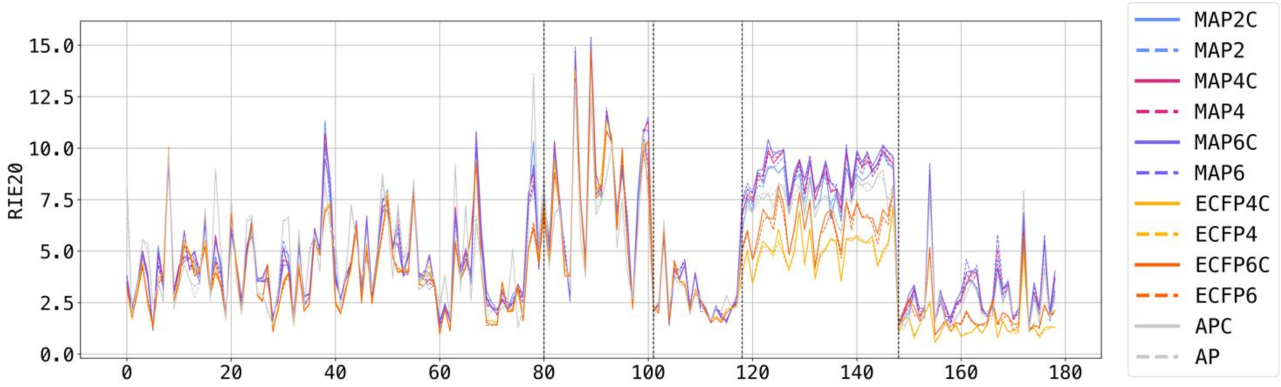

Figure S8. RIE20 of MAP2 (blue), MAP4 (magenta), MAP6 (purple), AP (grey), ECFP4 (yellow) and ECFP6 (orange) across all small molecules and peptide targets (80 ChEMBL targets, 21 DUD targets, 17 MUV targets, 30 mutated peptide targets, and 30 scrambled peptide targets). Chiral fingerprints are displayed as bold lines, non-chiral fingerprints are displayed as dashed lines. The value displayed for each dataset is the mean metric of 5 runs.

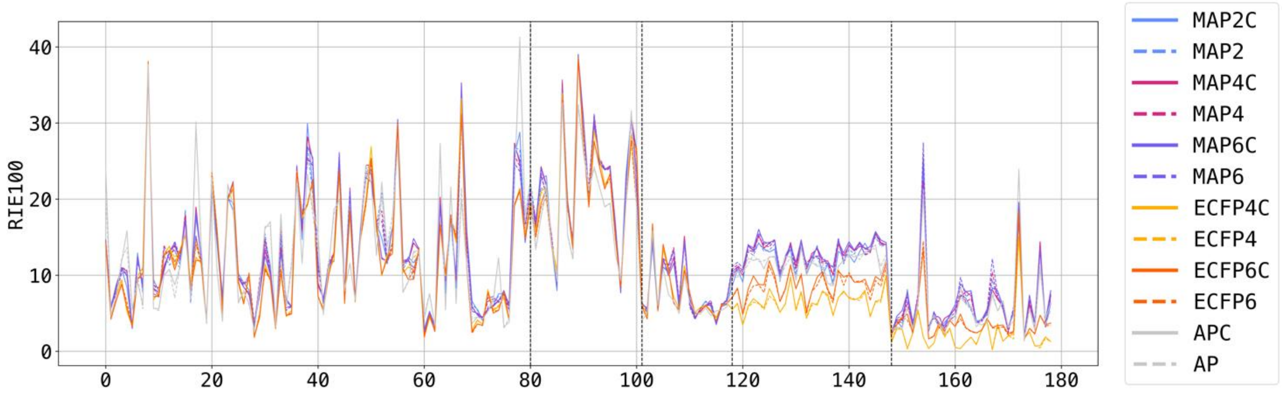

Figure S9. RIE100 of MAP2 (blue), MAP4 (magenta), MAP6 (purple), AP (grey), ECFP4 (yellow) and ECFP6 (orange) across all small molecules and peptide targets (80 ChEMBL targets, 21 DUD targets, 17 MUV targets, 30 mutated peptide targets, and 30 scrambled peptide targets). Chiral fingerprints are displayed as bold lines, non-chiral fingerprints are displayed as dashed lines. The value displayed for each dataset is the mean metric of 5 runs.

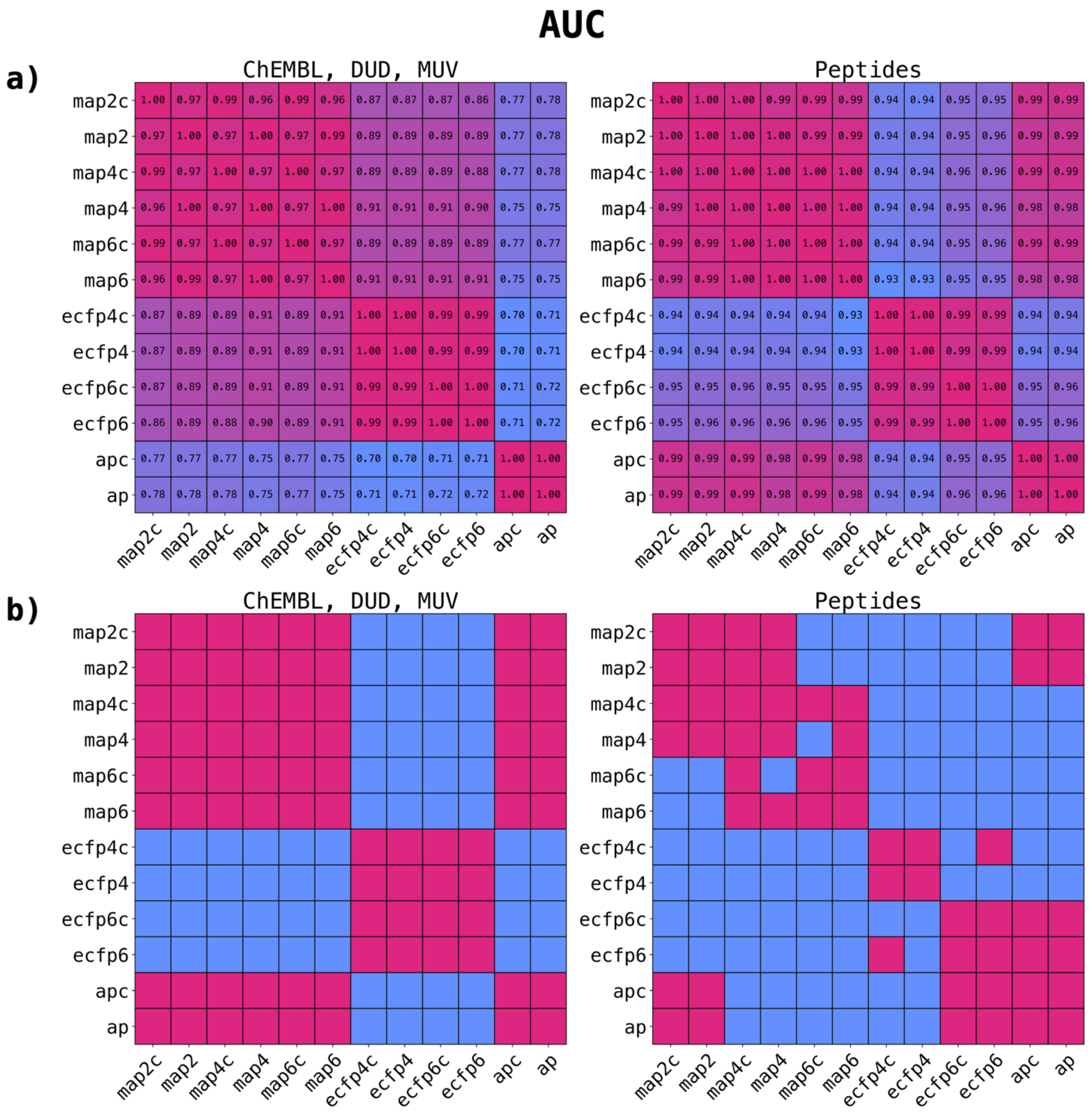

Figure S10: **a)** Pairwise Pearson correlations among tested fingerprints, derived from the mean AUCs acquired from benchmark datasets. The numbers represent the Pearson correlation coefficient for each pair. **b)** Pairwise Friedman-Nemenyi test among tested fingerprints, based on the ranked AUCs from benchmark datasets. A red square denotes a not significant difference between fingerprints at α=0.05, while a blue square denotes a significant difference.

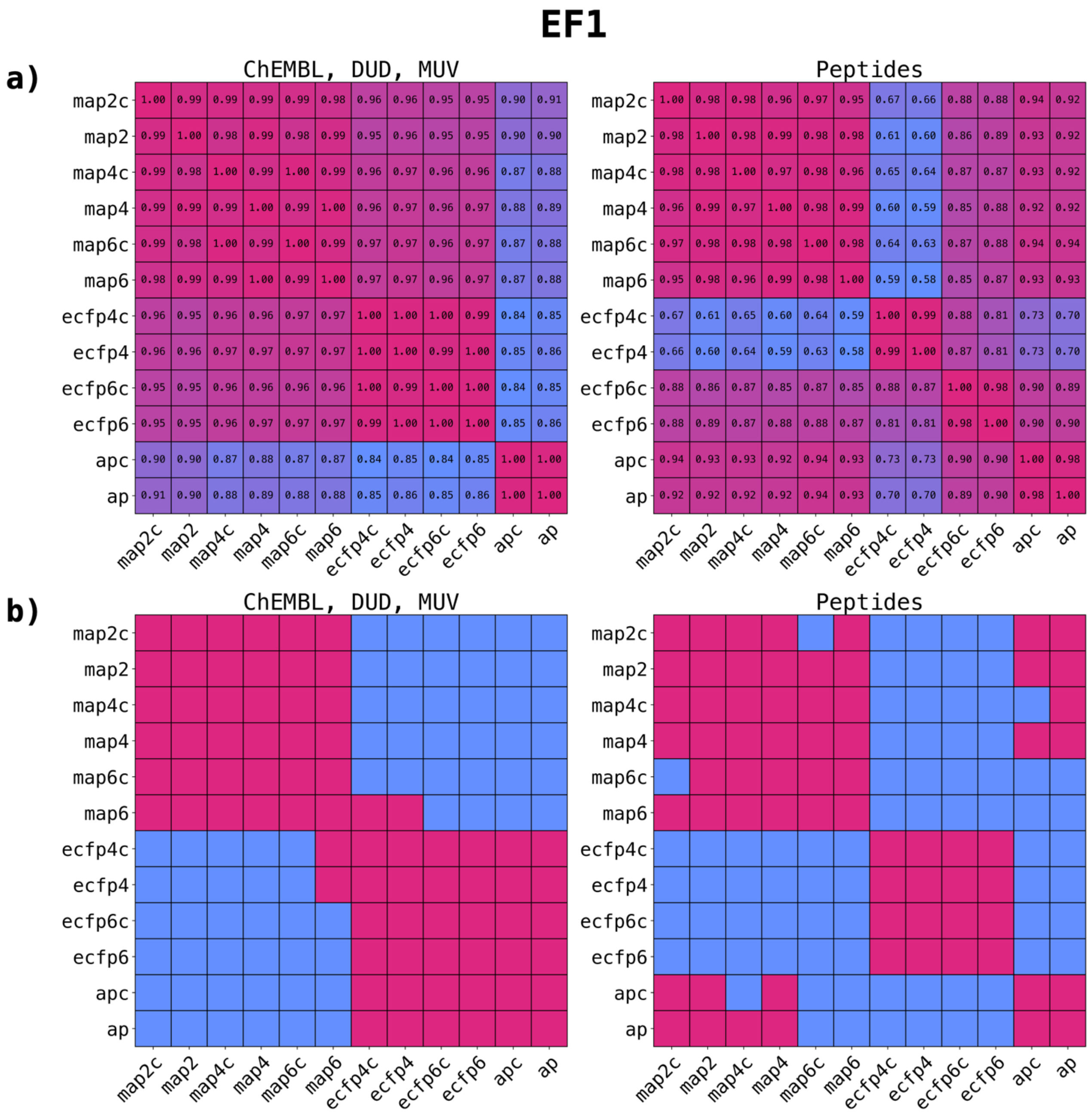

Figure S11: **a)** Pairwise Pearson correlations among tested fingerprints, derived from the mean EF1s acquired from benchmark datasets. The numbers represent the Pearson correlation coefficient for each pair. **b)** Pairwise Friedman-Nemenyi test among tested fingerprints, based on the ranked EF1s from benchmark datasets. A red square denotes a not significant difference between fingerprints at α=0.05, while a blue square denotes a significant difference.

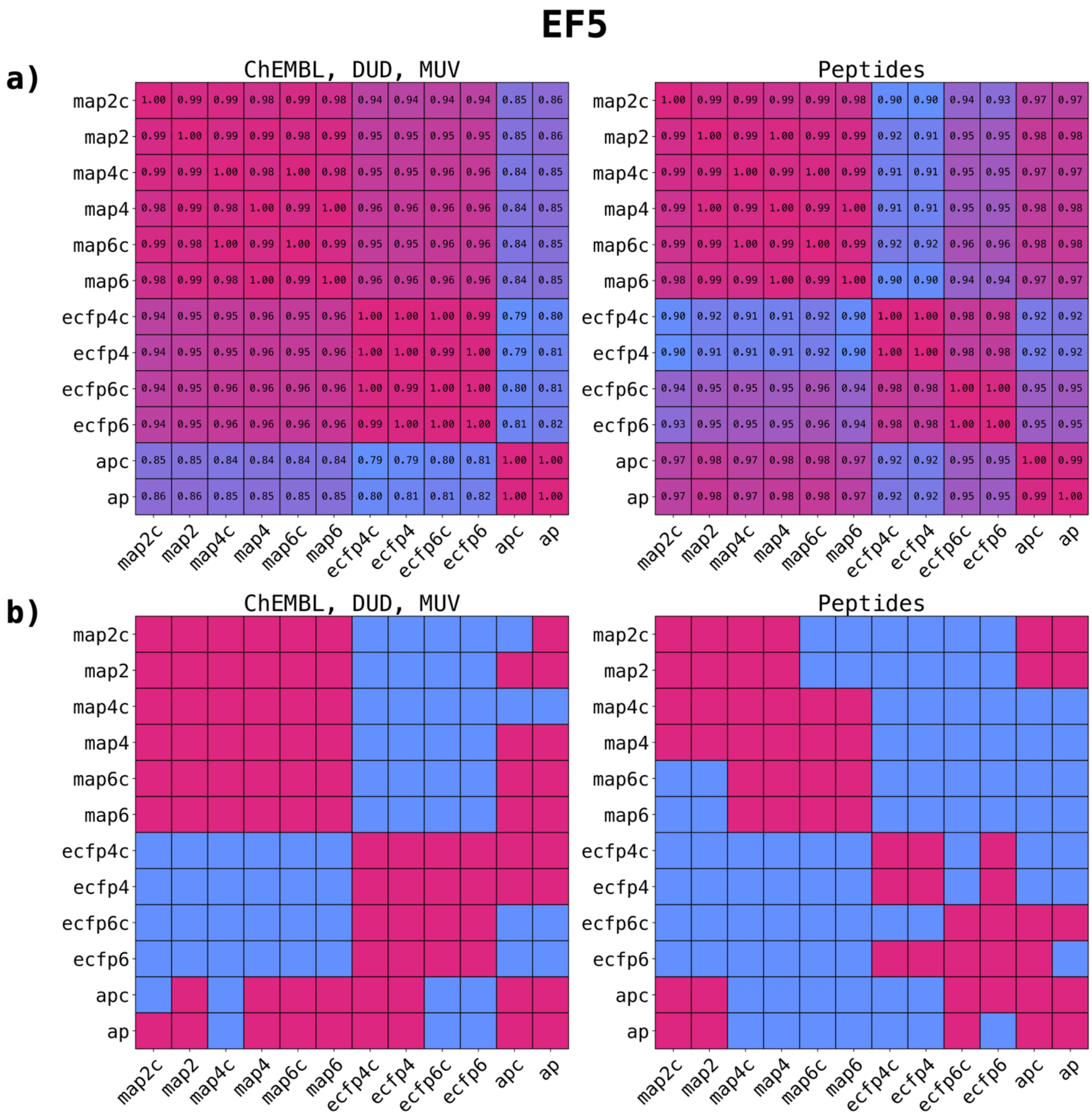

Figure S12: **a)** Pairwise Pearson correlations among tested fingerprints, derived from the mean EF5s acquired from benchmark datasets. The numbers represent the Pearson correlation coefficient for each pair. **b)** Pairwise Friedman-Nemenyi test among tested fingerprints, based on the ranked EF5s from benchmark datasets. A red square denotes a not significant difference between fingerprints at α=0.05, while a blue square denotes a significant difference.

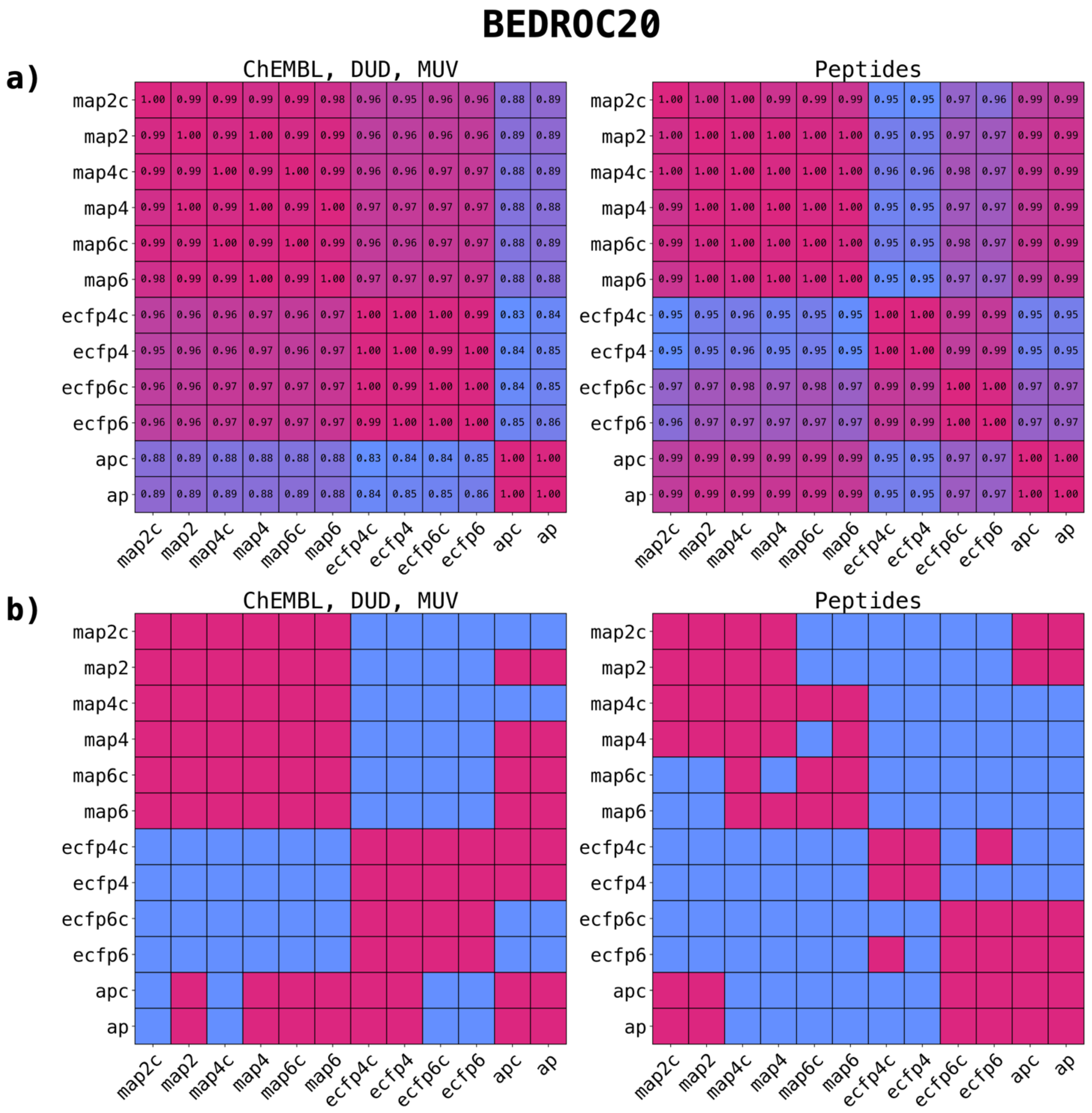

Figure S13: **a)** Pairwise Pearson correlations among tested fingerprints, derived from the mean BEDROC20s acquired from benchmark datasets. The numbers represent the Pearson correlation coefficient for each pair. **b)** Pairwise Friedman-Nemenyi test among tested fingerprints, based on the ranked BEDROC20s from benchmark datasets. A red square denotes a not significant difference between fingerprints at α=0.05, while a blue square denotes a significant difference.

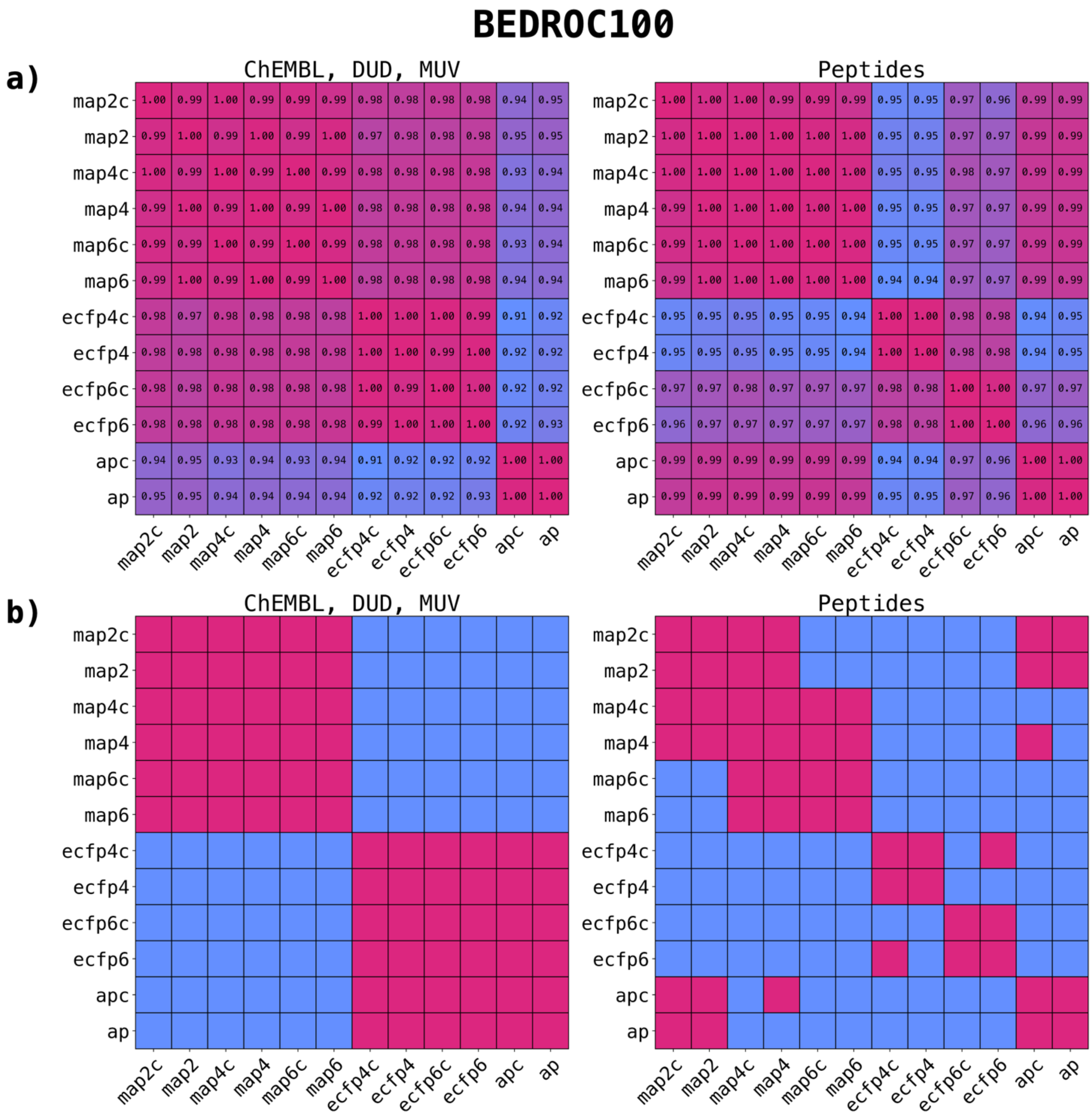

Figure S14: **a)** Pairwise Pearson correlations among tested fingerprints, derived from the mean BEDROC100s acquired from benchmark datasets. The numbers represent the Pearson correlation coefficient for each pair. **b)** Pairwise Friedman-Nemenyi test among tested fingerprints, based on the ranked BEDROC100s from benchmark datasets. A red square denotes a not significant difference between fingerprints at α=0.05, while a blue square denotes a significant difference.

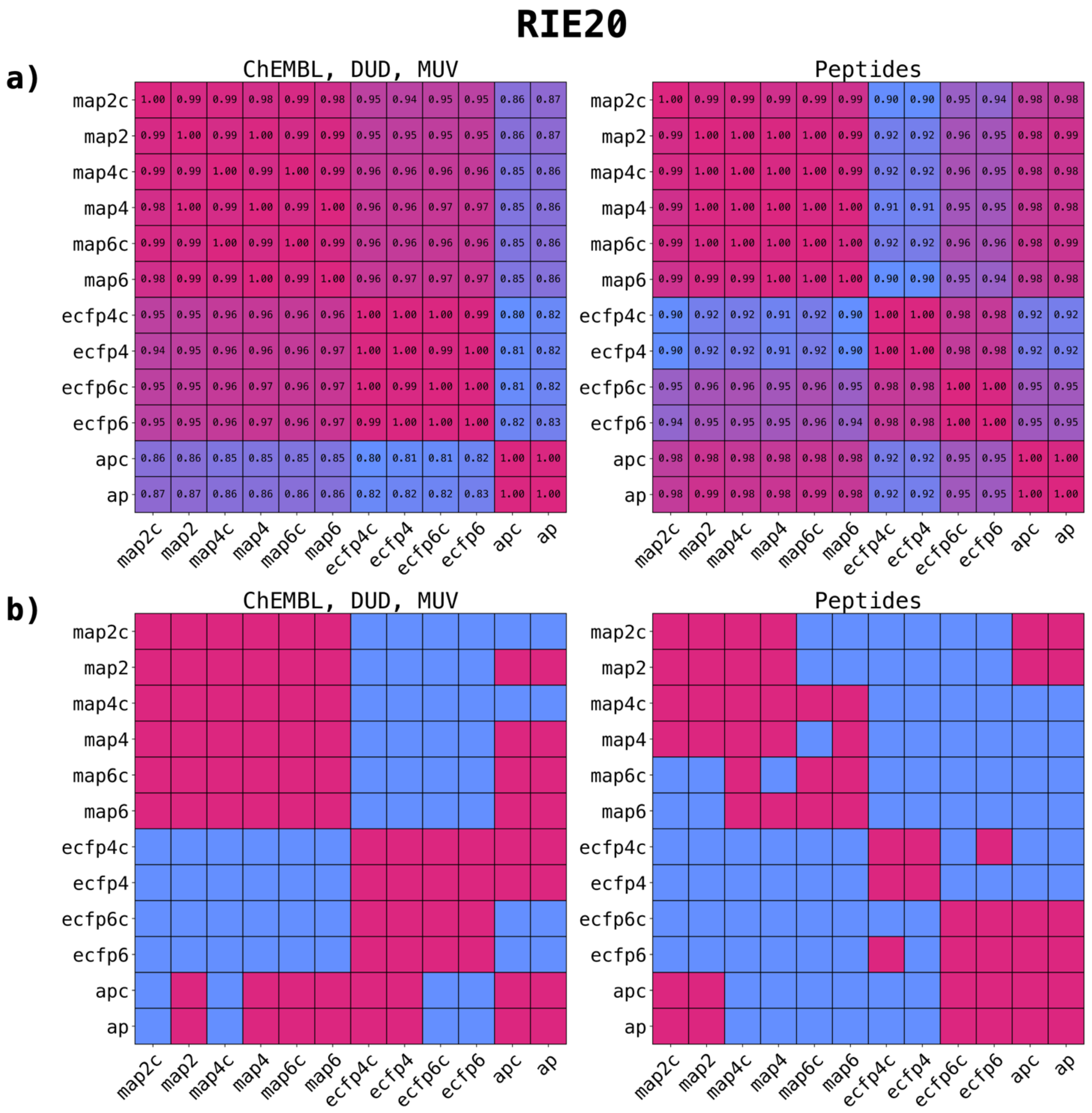

Figure S15: **a)** Pairwise Pearson correlations among tested fingerprints, derived from the mean RIE20s acquired from benchmark datasets. The numbers represent the Pearson correlation coefficient for each pair. **b)** Pairwise Friedman-Nemenyi test among tested fingerprints, based on the ranked RIE20s from benchmark datasets. A red square denotes a not significant difference between fingerprints at α=0.05, while a blue square denotes a significant difference.

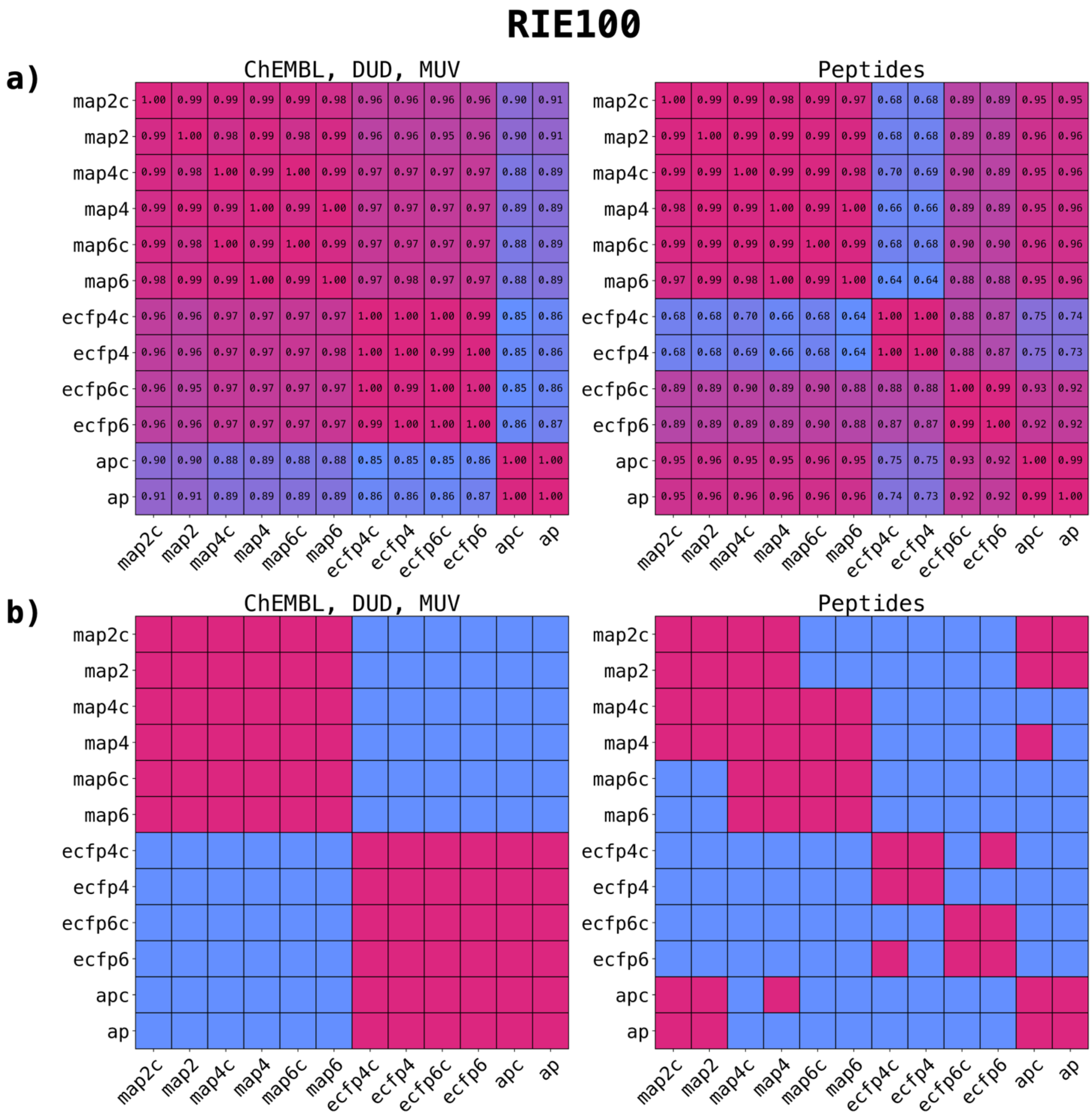

Figure S16: **a)** Pairwise Pearson correlations among tested fingerprints, derived from the mean RIE100s acquired from benchmark datasets. The numbers represent the Pearson correlation coefficient for each pair. **b)** Pairwise Friedman-Nemenyi test among tested fingerprints, based on the ranked RIE100s from benchmark datasets. A red square denotes a not significant difference between fingerprints at α=0.05, while a blue square denotes a significant difference.

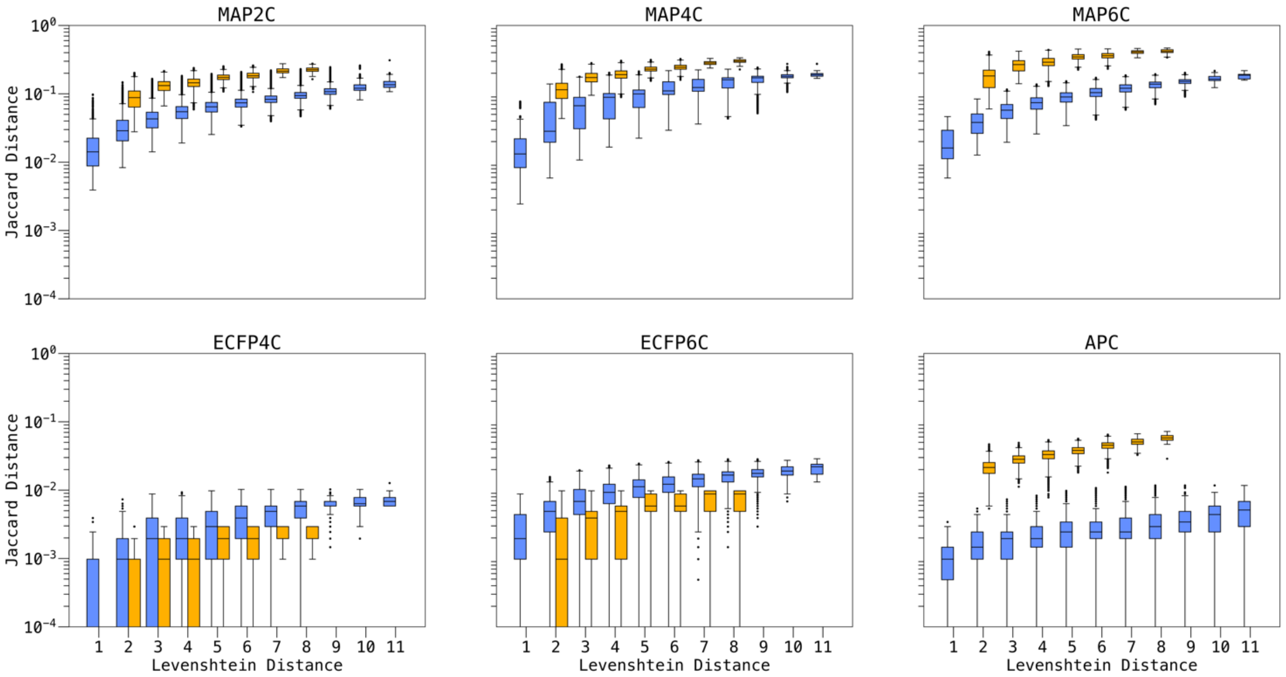

Figure S17. Comparative analysis of MAP2C, MAP4C, MAP6C, APC, ECFP4C and ECFP6C Jaccard distance assignment on ln65 diastereomers (blue) and structural isomers (yellow). The distance distributions are grouped by Levenshtein distance, used to determine the number of mutations from any sequence to ln65. MAPC fingerprints display a higher performance than the other fingerprints when it comes to distinguishing all possible diastereomers and structural isomers from each other. This is not the case for APC, which has difficulties distinguishing diastereomers, and ECPFC fingerprints, which cannot distinguish diastereomers or structural isomers robustly. MAPC fingerprints also consistently assign lower distances to diastereomers than structural isomers. APC follows the same trend, although the lower diastereomer distances are skewed due to the APC fingerprint not being able to robustly distinguish all diastereomers. ECFPC show a complete overlap of Jaccard distances for diastereomers and structural isomers. Finally, the overall Jaccard distances increase with increasing Levenshtein distance for MAPC fingerprints, indicating that the obtained distances align with intuitive changes such as stereocenter or residue mutations.

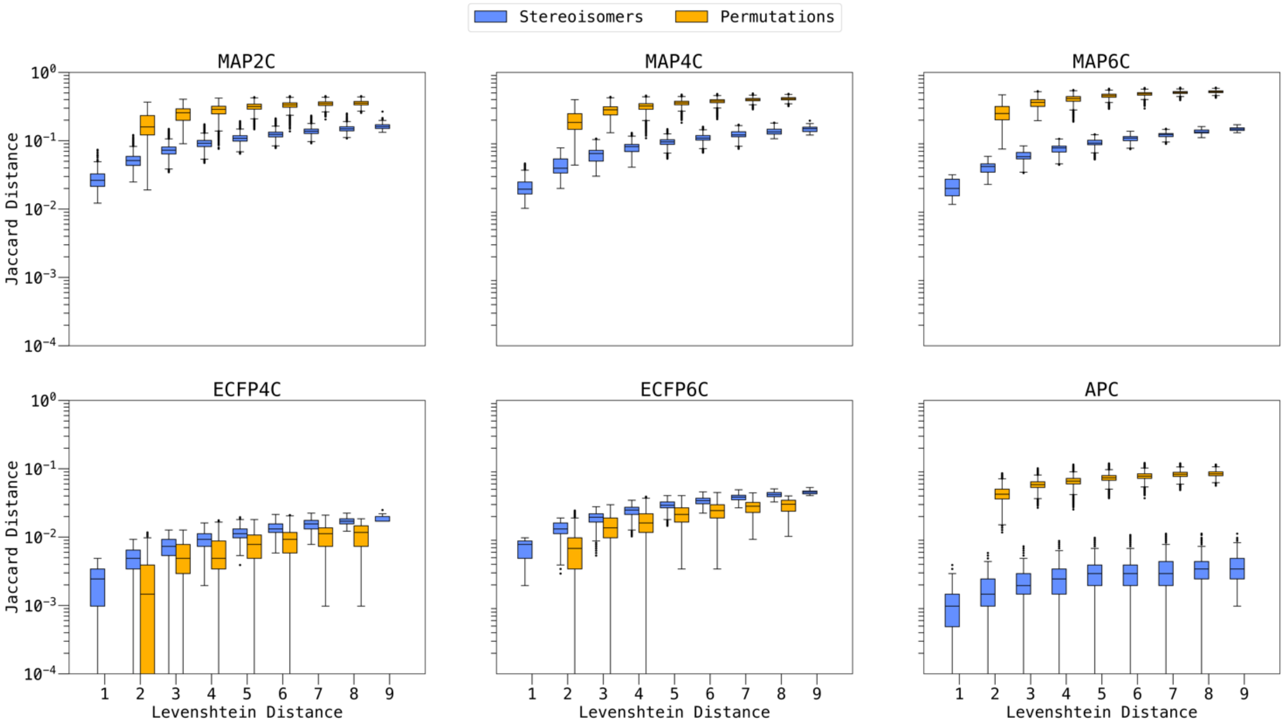

Figure S18. Comparative analysis of MAP2C, MAP4C, MAP6C, APC, ECFP4C and ECFP6C Jaccard distance assignment on polymyxin B2 diastereomers (blue) and structural isomers (yellow). The distance distributions are grouped by Levenshtein distance, used to determine the number of mutations from any sequence to polymyxin B2. MAPC fingerprints display a higher performance than the other fingerprints when it comes to distinguishing all possible diastereomers and structural isomers from each other. This is not the case for APC, which has difficulties distinguishing diastereomers, and ECPFC fingerprints, which cannot distinguish diastereomers or structural isomers robustly. MAPC fingerprints also consistently assign lower distances to diastereomers than structural isomers. APC follows the same trend, although the lower diastereomer distances are skewed due to the APC fingerprint not being able to robustly distinguish all diastereomers. ECFPC show a complete overlap of Jaccard distances for diastereomers and structural isomers. Finally, the overall Jaccard distances increase with increasing Levenshtein distance for MAPC fingerprints, indicating that the obtained distances align with intuitive changes such as stereocenter or residue mutations.
